# Delineation of polar localization domains of *Agrobacterium tumefaciens* type IV secretion apparatus proteins VirB4 and VirB11

**DOI:** 10.1002/mbo3.208

**Published:** 2014-09-13

**Authors:** Aditi Das, Anath Das

**Affiliations:** 1 Roseville Area High SchoolRoseville, Minnesota, 55113; 2 Department of Biochemistry, Molecular Biology and Biophysics, University of MinnesotaMinneapolis, Minnesota, 55455

**Keywords:** Cell pole, polar localization domain, type IV secretion, VirB proteins, virulence

## Abstract

*Agrobacterium tumefaciens* transfers DNA and proteins to a plant cell through a type IV secretion apparatus assembled by the VirB proteins. All VirB proteins localized to a cell pole, although these conclusions are in dispute. To study subcellular location of the VirB proteins and to identify determinants of their subcellular location, we tagged two proteins, VirB4 and VirB11, with the visual marker green fluorescent protein (GFP) and studied localization of the fusion proteins by epifluorescence microscopy. Both GFP-VirB4 and GFP-VirB11 fusions localized to a single cell pole. GFP-VirB11 was also functional in DNA transfer. To identify the polar localization domains (PLDs) of VirB4 and VirB11, we analyzed fusions of GFP with smaller segments of the two proteins. Two noncontiguous regions in VirB4, residues 236–470 and 592–789, contain PLDs. The VirB11 PLD mapped to a 69 amino acid segment, residues 149–217, in the central region of the protein. These domains are probably involved in interactions that target the two proteins to a cell pole.

## Introduction

*Agrobacterium tumefaciens*, a soil bacterium pathogenic to most plants, is the causative agent of the cancerous “Crown Gall” tumor disease (Zupan et al. 2000). Crown gall disease particularly attacks graft junction in fruit trees and grapevines, and causes injuries at the ground level (“crown”) in other plants. Tumors result from the stable transfer and integration of a segment of bacterial plasmid DNA, the T-DNA, into the plant nuclear genome. T-DNA transfer to plants requires the virulence (*vir*) region of the tumor inducing (Ti-) plasmid. The *vir* region encompasses ∼30 kilobase (kb) of DNA and encodes more than 20 proteins. Eighteen of the Vir proteins are essential for DNA transfer. These proteins process the T-DNA and assemble a passageway at the bacterial membranes for the transfer of the T-strand DNA intermediate to the plant cell. VirD4 and 11 VirB proteins participate in the function and assembly of a type IV secretion apparatus and a T-pilus used for DNA transfer (Zupan et al. 2000; Christie et al. 2005). Type IV secretion is used by bacteria for the transfer of conjugal plasmids, and macromolecules that affect host cellular processes causing diseases in humans and animals, for example, whooping cough (*Bordetella pertussis*), peptic ulcer and cancer (*Helicobacter pylori*), Legionnaires’ disease (*Legionella* sp.), and brucellosis (*Brucella* sp.) (Llosa et al. 2009).

*Agrobacterium tumefaciens* assembles the DNA transfer apparatus at a cell pole (Judd et al. 2005a). Analysis by immunofluorescence microscopy showed that the VirB proteins and VirD4 localize to a cell pole (Kumar and Das 2002; Judd et al. 2005a,b). VirD4 and four VirB proteins, VirB3, VirB4, VirB8, and VirB11, do not require other Vir proteins for their polar localization. VirB7, VirB9, and VirB10, on the other hand, localized to a cell pole only when VirB8 was present suggesting that VirB8 targets these proteins to the cell pole (Judd et al. 2005a). Recent studies from another laboratory suggested that the VirB proteins localize throughout the cell surface (Aguilar et al. 2010, 2011). Fusions of GFP and VirB proteins were found distributed in a helical array throughout the cell circumference. Other studies reported that the coupling protein VirD4 targets a substrate of the *Agrobacterium* DNA transfer system, VirE2, to a cell pole, and other proteins and structures associated with T-DNA transfer, viz., VirC1, a VirD2 binding protein VBP and the T-pilus, localize to a cell pole (Lai et al. 2000; Atmakuri et al. 2003, 2007; Guo et al. 2007).

Many proteins and large protein complexes in bacteria are found at the cell poles. Several type IV secretion apparatus proteins from diverse bacteria are also found at a cell pole. Proteins of the *Bacillus subtilis* conjugative element ICEBS1, *Clostridium perfringens* plasmid conjugation system, and the *Legionella pneumophila* type IV secretion apparatus have been localized to the cell poles (Teng et al. 2008; Berkman et al. 2010; Vincent et al. 2012). A cell pole is used for the attachment of *Bradyrhizobium* to its host plant root hairs and that of *Agrobacterium* to the host plant cell (Matthysse 1987; Loh et al. 1993; Brown et al. 2012). It has been suggested that the lateral surface of *Agrobacterium*, and not the cell pole, is involved in the attachment to plant cells (Aguilar et al. 2011).

Some proteins are found at a specific pole of only one cell type, for example, the *Shigella flexneri* IscA is found at the old pole and *Caulobacter* PodJ is found at a pole of the swarmer cell (Charles et al. 2001; Hinz et al. 2003). The *Escherichia coli* chemotaxis machinery is found at a cell pole (Maddock and Shapiro 1993). Surface structures like flagella, pili, and stalks are preferentially assembled at a cell pole (Dworkin 2009; Bowman et al. 2011).

The *Agrobacterium* VirB6–VirB10 proteins are the major constituents of the type IV secretion apparatus. Interactions among these proteins are required for the assembly of the type IV secretion apparatus (Christie et al. 2005). The VirB7, VirB9, and VirB10 homologs of pKM101, TraN, TraO, and TraF, assemble a large 1.05 MDa complex (Fronzes et al. 2009). The complex consisting of 14 units of the three proteins spans both membranes forming the core translocation channel. VirD4 and two VirB proteins, VirB4 and VirB11, provide energy for substrate translocation (Atmakuri et al. 2004). VirB4 is an inner membrane protein with four potential trans-membrane domains and large hydrophilic regions, and VirB11 is a peripheral membrane protein with no discernable membrane spanning domain (Dang and Christie 1997; Rashkova et al. 1997).

Green fluorescent protein (GFP) is widely used to study subcellular location of a protein (Zhang et al. 2002). GFP attached to a cytoplasmic domain of a protein fluoresces green when exposed to blue light. We used GFP fusions to determine the subcellular location of *Agrobacterium* VirB4 and VirB11 proteins. The two proteins were chosen because of their large hydrophilic regions with either small, defined hydrophobic regions (VirB4) or no recognized hydrophobic region (VirB11). We used deletion methods and cloning of defined gene segments to identify the regions required for the targeting of the two proteins to their appropriate subcellular location.

## Materials and Methods

### Construction of *gfp*-*virB4* and *gfp-virB11* fusion genes

Plasmid pADI15 containing a *gfp*-*virB4* fusion gene was constructed by cloning the *virB4* coding region sequences into plasmid vector pAD2005. The *virB4* coding region (codon 2-end) was isolated as a SacI fragment from plasmid pAD1885 (Mossey et al. 2010). Plasmid pAD2005 contains *gfp* under the control of the inducible *virD* promoter. It was constructed by cloning the *virD*_p_-*gfp* mut3 gene from plasmid pGFP3 into plasmid pAD1930. Plasmid pGFP3 is similar to pGFP2 (Mossey et al. 2010) except that the *virD*_p_-*gfp* gene was inserted in the other orientation. Plasmid pAD1930 is a wide host plasmid derived from the kanamycin-resistant colE1 plasmid vector pUK21. It was constructed by cloning a 3.2 kb HindIII-NcoI fragment from plasmid pTJS75 containing the *ori*V, *ori*T, and *trfA* regions of the IncP plasmid RK2 into plasmid pUK21 (Schmidhauser and Helinski 1985; Vieira and Messing 1991). Plasmid pADI15 was introduced into *Agrobacterium* A348ΔB4 by electroporation. *Agrobacterium* A348ΔB4 has a nonpolar deletion in *virB4* (Berger and Christie 1994).

The *virB11* coding region was obtained by PCR amplification and cloned as a BamHI-Acc65I fragment into plasmid pAD2005 to construct plasmid pADI16. Recognition sequences for the two enzymes were incorporated into the PCR primers (Table S1). Plasmid pADI16 was introduced into the *virB11* deletion mutant *Agrobacterium* A348ΔB11 (Berger and Christie 1994).

### Construction of *gfp*-*virB4*/*virB11* fusions with a specific segment of the *virB4*/*virB11* gene

The *virB4* gene in plasmid pADI15 contains two in-frame *Eco*RI and two in-frame *Bam*HI sites. Two deletions within *virB4* were made by deleting the 1.4 kb EcoRI fragment (pADI15ΔR) or the 0.7 kb BamHI fragment (pADI15ΔB). All other derivatives of *gfp*-*virB4* and all derivatives of *gfp*-*virB11* were constructed by cloning the desired fragment obtained by PCR amplification. Primers used for PCR amplification are listed in Table S1. The PCR amplified fragments were cloned as a BamHI-Acc65I fragment into plasmid vector pAD2005. All plasmids were confirmed by DNA sequence analysis.

### Epifluorescence microscopy

Bacteria were grown overnight at 30°C in AB medium containing antibiotic (50 *μ*g/mL kanamycin). To induce expression of a fusion gene, 5 mL AB induction medium (AB Mes, pH 5.8, 50 *μ*mol/L acetosyringone, and 50 *μ*g/mL kanamycin) was inoculated with 0.35 mL of the overnight culture and grown for 16–18 h at room temperature (Mossey et al. 2010). Seven microliters of induced cells were placed on a polylysine-coated glass slide, and a cover glass was placed on top. After 10 min on bench top to allow bacteria to adhere, samples were viewed with a Leica fluorescence microscope equipped with a Hamamatsu ORCA-ER digital CCD camera. Images were recorded with Volocity software (PerkinElmer, Waltham, MA) and processed using Adobe Photoshop CS2 for presentation. For quantitative analysis, number of labeled cells was counted and their fluorescence patterns were analyzed.

### Other methods

Accumulation of fusion proteins was monitored by electrophoresis on sodium dodecyl sulfate – 12.5% polyacrylamide gel. Proteins were transferred onto a nitrocellulose membrane and the membrane was probed overnight with anti-GFP, anti-VirB4, or anti-VirB11 antibodies. After incubation with Alexa 680 conjugated secondary antibodies, the filter was scanned using the Odyssey imaging system (Li-Cor Biosciences, Lincoln, NE). *Kalanchöe daigremontiana* leaves were used for tumor formation assays (Mossey et al. 2010). Tumors were scored 3 weeks after infection.

## Results

### Subcellular location of GFP-VirB4 and GFP-VirB11

**Figure 1:**
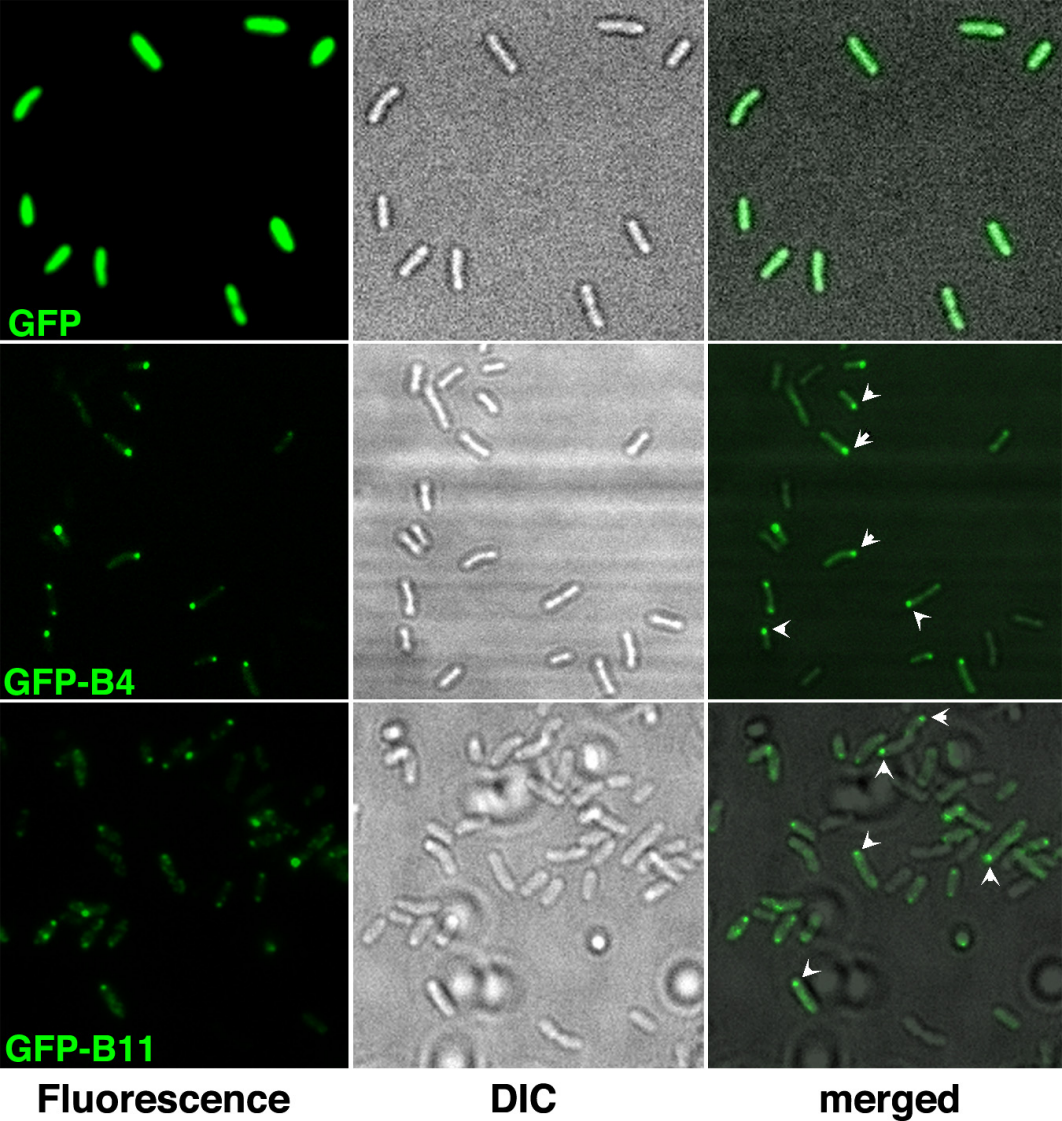
Subcellular localization of GFP-VirB4 and GFP-VirB11. Subcellular location of GFP and its fusions with VirB4 or VirB11 was determined by epifluorescence microscopy. Left panels, fluorescence microscopy; middle, light microscopy; right, both together. GFP, GFP alone; GFP-B4, GFP-VirB4; GFP-B11, GFP-VirB11. A subset of polar foci is identified with arrowheads.

A GFP tag was introduced at the N-terminus of VirB4 and VirB11 to facilitate analysis of subcellular location of the two proteins. The fusion genes are expressed from the inducible *virD* promoter on a wide host range RK2-derived plasmid. Plasmids pADI15 and pADI16 containing the *gfp*-*virB4* and *gfp*-*virB11* fusions were introduced into *Agrobacterium* A348ΔB4 and A348ΔB11, respectively. Analysis of subcellular location by epifluorescence microscopy showed that bacteria expressing GFP-VirB4 had localized foci of fluorescence (Fig.1, left panel, middle row). When cells were viewed with both fluorescence and light microscopy, almost all foci of fluorescence mapped to a cell end indicating that GFP-VirB4 localizes to a cell pole (right panel, middle row). The vast majority of the foci (92% of the polar foci, *n* = 189) mapped to a single cell pole while a few cells had fluorescence foci at both poles (Table1 and Fig.1). In control experiments, *Agrobacterium*-expressing GFP exhibited green fluorescence throughout the cell (left panel, top row).

**Table 1:** Quantitative analysis of subcellular localization of GFP-VirB4 and its derivatives.

VirB4 segment	Plasmid	Total number of cells	One pole	Both poles	Others	Total number at poles	% Polar
2–789	pADI15	191	173	16	2	189	99
2–315 + 781–789	pADI15ΔR	111	9	0	102	9	0.1
236–789	pADI15ΔB	152	122	16	14	138	91
236–470	pADI18	125	66	24	35	90	72
236–444	pADI33	209	5	1	203	6	3
275–470	pADI35	129	38	8	83	46	36
562–789	pADI20	190	118	23	49	141	74
592–789	pADI27	218	153	27	38	180	83
592–721	pADI28	149	15	6	128	21	14
592–755	pADI37	190	114	16	60	130	68
643–755	pADI38	187	6	1	180	7	4
643–789	pADI39	135	70	15	50	85	63

Number of bacteria with one polar focus, two polar foci, and other phenotypes (no foci, three and more foci, and nonpolar focus) were counted. Percent polar is defined as 100× (number of bacteria with one and two polar foci/number of fluorescent bacteria).

GFP-VirB11, like GFP-VirB4, formed foci of fluorescence (left panel, bottom row). Almost all foci mapped to a cell pole (right panel) indicating that GFP-VirB11, like GFP-VirB4, localized to a cell pole. Quantitative analysis showed that 99% (*n* = 191) of GFP-VirB4 foci and 76% (*n* = 154) of GFP-VirB11 foci localized to a cell pole (Tables1 and 2). Both VirB4 and VirB11, therefore, localized to a cell pole.

**Table 2:** Quantification of subcellular localization of GFP-VirB11 and its derivatives.

VirB11 segment	Plasmid	Total number of cells	One pole	Both poles	Others	Total number at poles	% Polar
2–343	pADI16	154	88	29	37	117	76
2–217	pADI23	364	210	62	92	272	75
149–343	pADI22	152	103	19	30	122	80
149–217	pADI30	131	67	14	50	81	62
149–248	pADI41	229	93	29	107	122	53
123–248	pADI40	209	154	23	32	177	85

Number of bacteria with one polar focus, two polar foci and other phenotypes were counted. Percent polar is defined as 100 × (number of bacteria with one and two polar foci/number of fluorescent bacteria).

**Figure 2:**
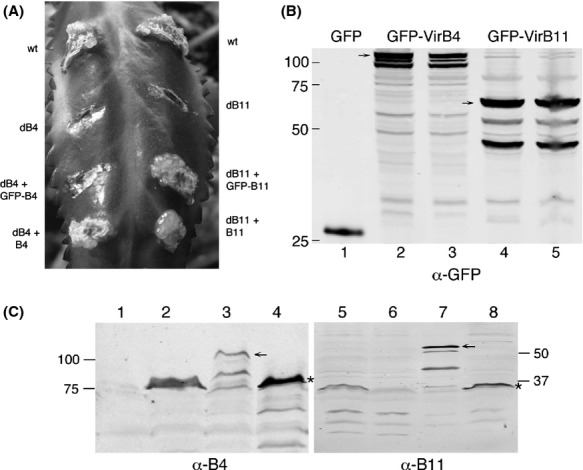
DNA transfer activity and accumulation of GFP-VirB4 and GFP-VirB11 fusion proteins. (A) The ability of the fusion proteins to support tumor formation on *Kalanchoe* leaf was determined. Leaves were infected with the strains indicated. wt, wild-type *Agrobacterium* A348; dB4, A348ΔB4; dB11, A348ΔB11; GFP-B4, pADI15; GFP-B11, pADI16; B4, pvirB4; B11, pVirB11. (B,C) Accumulation of GFP-VirB4 and GFP-VirB11 was monitored by western blot assay following SDS-PAGE. Blots were probed with anti-GFP, anti-VirB4 or anti-VirB11 antibodies. An arrow identifies the full-length fusion protein. Numbers on left/right indicate the molecular mass (kDa) of marker proteins. The VirB4/VirB11-specific band in Figure2C is marked with an asterisk. (B) Lane 1, GFP; lanes 2 and 3, GFP-VirB4; lanes 4 and 5, GFP-VirB11. (C) Lanes 1 and 6, uninduced A348; lanes 2 and 5, induced A348; lane 3, GFP-VirB4; lanes 4 and 8, A348ΔB/pvirB; lane 7, GFP-VirB11.

The effect of addition of GFP on DNA transfer function of VirB4 and VirB11 was monitored by complementation assays (Fig.2A). Bacteria with GFP-VirB4 did not complement a nonpolar deletion in *virB4* indicating that the addition of GFP at the VirB4 N-terminus is detrimental to VirB4 function. The GFP-VirB11 fusion, on the other hand, was fully functional as it complemented the deletion in the cognate *virB11* gene. VirB11 thus can tolerate additional sequences at its N-terminus. The two fusion proteins had no negative effect on the tumor-forming ability of wild-type *Agrobacterium* A348 and both fusion proteins accumulated in the cell although some degradation of both fusions was observed (Fig.2B and data not shown). Accumulation of GFP-VirB4 was comparable to that of VirB4 in wild-type bacteria and that of GFP-VirB11 was comparable to that in bacteria with *virB11* on a low copy plasmid (Fig.2C).

### Mapping the polar localization domain of VirB4

VirB4 is a large protein containing 789 residues. We used deletion methods and cloning of defined segments to identify the minimum sequences required for polar localization of VirB4. We introduced two deletions in the *gfp*-*virB4* fusion in plasmid pADI15 by removing an EcoRI fragment that deleted *virB4* codons 316–780 (plasmid pADI15ΔR), and a BamHI fragment that deleted *virB4* codons 1–235 (pADI15ΔB).

**Figure 3:**
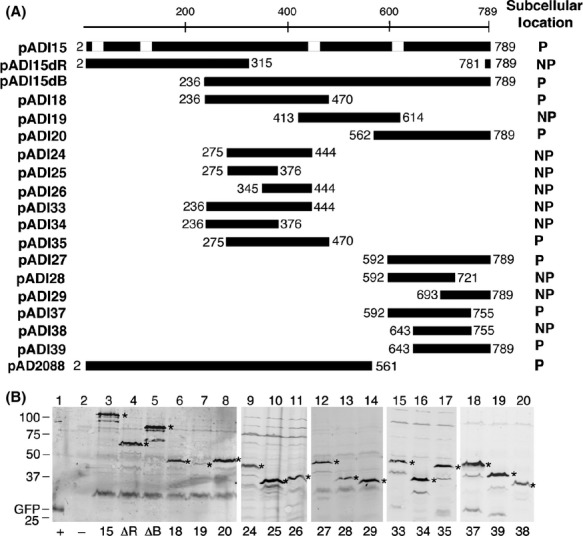
Identification of VirB4 polar localization domain. (A) GFP-VirB4 fusions constructed in this study and the subcellular locations of the fusion proteins are shown. The VirB4 region at the C-terminus of GFP is shown as a dark box. Numbers indicate the *virB4* codons present in the fusion. The white boxes on the full-length VirB4 map (pADI15) indicate locations of the four hydrophobic regions that can function in membrane spanning (Dang and Christie 1997). P, polar; NP, nonpolar. (B) Expression of GFP-VirB4 fusion proteins. Accumulation of GFP-VirB4 and its derivatives were monitored as described in legend to Figure2B. The full-length fusion proteins are marked with an asterisk. Accumulation of degradation products migrating below the major band was noted in some samples (lanes 3, 5, 10, and 15–19). Numbers below the figure identify the pADI plasmid the strain harbors. ΔR, pADI15ΔR; ΔB, pADI15ΔB; +, bacteria expressing GFP alone; –, bacteria with no GFP. Numbers on left indicate the molecular mass (kDa) of marker proteins. Protein encoded by plasmid pADI19 (lane 7) accumulated at a low level.

**Figure 4:**
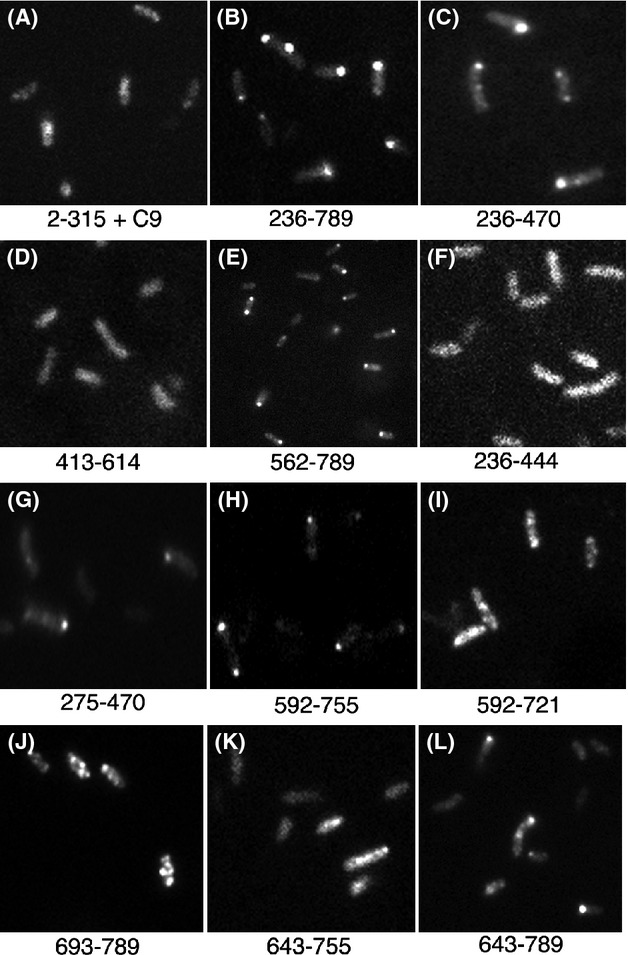
Localization of GFP-VirB4 fusions. Subcellular location of representative fusions of GFP and VirB4 segments (residue numbers below the panel) are shown. The fusion in panel A contains VirB4 residues 2–315 and nine residues from the C-terminus (C9).

Analysis by epifluorescence microscopy showed that bacteria harboring plasmid pADI15ΔR had fluorescence throughout the cell while bacteria with pADI15ΔB formed polar foci (Figs.3, 4A and B). The N-terminal end of VirB4, therefore, is not required for its polar localization, and amino acids 236–789 contain the VirB4 polar localization domain (PLD). Protein analysis by western blot assays showed that both fusion proteins accumulated at a comparable level (Fig.3B, lanes 4 and 5).

To better define the VirB4 PLD, we analyzed three *gfp* fusions containing *virB4* codons 236–470, 413–614, and 562–789 (encoded by plasmids pADI18, pADI19, and pADI20, respectively; Fig.3). Two fusion proteins, GFP-VirB4_236-470_ and GFP-VirB4_562-789_, were found predominantly at a cell pole as bacteria expressing the two proteins had fluorescent foci at the cell poles (Fig.4C and E; Table1). The third protein, GFP-VirB4_413-614_, was found distributed throughout the cell, however, bacteria expressing this fusion exhibited a low level of fluorescence (Fig.4D). Analysis by western blot showed that GFP-VirB4_413-614_ accumulated at a very low level in the cell (Fig.3, lane 7). These results suggest that at least two nonoverlapping regions, residues 236–470 (region R1) and 562–789 (region R2), contain information required for the polar targeting of VirB4.

To identify the minimal PLD sequences, we constructed additional fusions by cloning defined DNA fragments generated by PCR amplification. Only one region R1 fusion, GFP-VirB4_275-470_, exhibited a low affinity for the cell poles (36% polar, Table1; Fig.4G). Five other fusions within this region localized throughout the bacterium and showed no preference for a cell pole (Fig.3).

Six fusions were analyzed to identify the PLD within region R2 (plasmids pADI27-29 and pADI37-39, Fig.3). Three fusion proteins, GFP-VirB4_592-789_, GFP-VirB4_592-755_, and GFP-VirB4_643-789_, localized to a cell pole (Table1 and Fig.4H and L). Bacteria with two fusions, GFP-VirB4_592-721_ and GFP-VirB4_693-789_, had multiple foci of fluorescence distributed throughout the cell periphery (Fig.4I and J). GFP-VirB4_592-721_, however, localized to a cell pole at a very low frequency (14% polar, Table1). The sixth fusion protein, GFP-VirB4_643-755_, was found distributed throughout the cell (Fig.4K; Table1).

Both GFP-VirB4_592-755_ and GFP-VirB4_643-789_ localized to a cell pole at a high efficiency (63–68% polar, Table1). Polar targeting efficiency of the two fusions was slightly lower than that of the larger fusions, GFP-VirB4_592-789_ and GFP-VirB4_562-789_. Therefore, VirB4 residues 643–755 are likely to contain the PLD. The GFP-VirB4_643-755_ fusion, however, did not localize to a cell pole. A plausible explanation for this observation is that sequences at either end of residues 643–755 stabilize a domain structure required for polar targeting.

### Mapping PLD of VirB11

**Figure 5:**
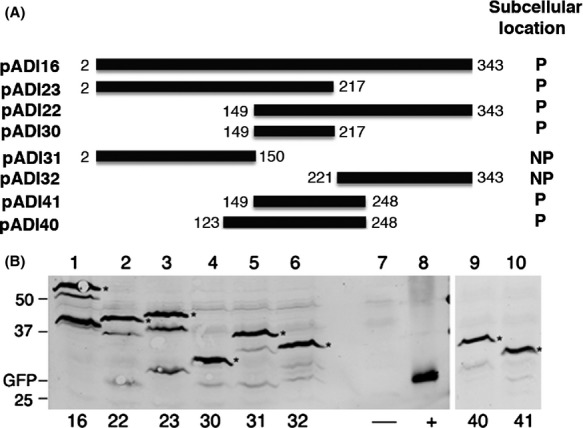
Analysis of GFP-VirB11 and its derivatives. (A) The *gfp*-*virB11* fusions constructed in this study and subcellular locations of the encoded fusion proteins are shown. P, polar; NP, nonpolar. (B) Accumulation of fusion proteins was analyzed by western blot assays as described in legend to Figure3. Numbers below the figure identify the pADI plasmid in the strain analyzed. The lower band(s) seen in some lanes are degradation products (*, fusion protein). −, no GFP; +, GFP alone.

**Figure 6:**
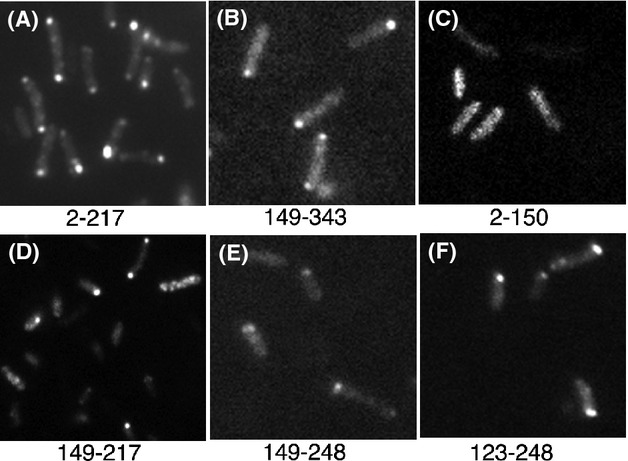
Subcellular localization of GFP-VirB11 derivatives. Subcellular locations of selected GFP-VirB11 fusion proteins are shown. Numbers indicate the VirB11 residues present in the fusion.

To map the PLD of VirB11, we first analyzed two fusions that express GFP-VirB11_2-217_ and GFP-VirB11_149-343_ (Fig.5A). Both fusion proteins accumulated at a comparable level in the cell, although GFP-VirB11_2-217_ and GFP-VirB11 accumulated a significant amount of degradation products (Fig.5B, lanes 1 and 3). Analysis by epifluorescence microscopy showed that both GFP-VirB11_2-217_ and GFP-VirB11_149-343_ localized to a cell pole at a similar efficiency (Fig.6A and B, and Table2). Therefore, VirB11 residues common in the two fusion proteins, residues 149–217, probably encode the PLD.

To test this hypothesis, five additional fusions were analyzed. Two fusion proteins, GFP-VirB11_2-150_ and GFP-VirB11_221-343_, were found distributed throughout the cell (Figs.5A and 6C). The other three fusions, GFP-VirB11_149-217_, GFP-VirB11_149-248_, and GFP-VirB11_123-248_, all localized to the cell poles (Fig.6D–F, and Table2). The GFP fusion with the smallest VirB11 segment, residues 149–217, localized to the cell poles at a high efficiency (62% polar, Table2) supporting the hypothesis that this region is sufficient for polar targeting of VirB11. An additional 31 C-terminal VirB11 residues (residues 218–248) had a small effect on the efficiency of polar targeting. However, a similar addition at the N-terminus (residues 123–148) significantly improved efficiency of polar targeting (85% polar). VirB11 residues 149–217 thus contain all information necessary for its targeting to a cell pole and residues 123–217 are sufficient for quantitative targeting.

## Discussion

Our study demonstrates that GFP fusions of *Agrobacterium* VirB4 and VirB11 proteins localize to a cell pole (Fig.1; Tables 1 and 2). These results are in agreement with earlier studies that found all the VirB proteins at a cell pole in wild-type *Agrobacterium* (Judd et al. 2005a,b). We also found that the GFP-VirB11 fusion was fully functional in DNA transfer and the GFP-VirB4 was not (Fig.2). The addition of GFP at the N-terminus of VirB4 thus affected its DNA transfer function but not the polar localization function. The N-terminal 235 residues of VirB4 are not required for its polar localization (Fig.3).

Two noncontiguous regions in VirB4, R1 (residues 236–470) and R2 (residues 592–789), could independently target a GFP fusion to a cell pole. Whether both regions are required for polar localization in the native protein is not known. For efficient polar targeting, region R1 is required in its entirety as small deletions at either end of region R1 led to either a significant reduction in or a complete loss of polar targeting (Table1, Fig.4). In region R2, residues 643–755 are necessary, but not sufficient, for polar localization of VirB4. GFP-VirB4_643-755_ was found distributed throughout the cell (Table1). The addition of 45–50 residues at the either end of this region (GFP-VirB4_592-755_ and GFP-VirB4_643-789_), however, led to a large increase in polar targeting (∼65% polar, Table1). We postulate that the additional residues at either end stabilize a structure of the central region required for polar targeting of VirB4. The PLD of VirB11 was mapped to a small 69 amino acid region encompassing residues 149–217 (Fig.5, Table2). The addition of 26 N-terminal residues led to an increase in polar targeting probably through the stabilization of protein structure. The VirB11 family of proteins is composed of two domains formed by the N-terminal and C-terminal halves of the protein (Yeo et al. 2000). The VirB11 PLD is at the N-terminal end of the CTD and includes the ATP binding domain (Walker A motif at residues 169–176). Polar targeting of VirB11, however, does not require a functional Walker A motif (Judd et al. 2005a).

Our results are in agreement with several studies (Atmakuri et al. 2003, 2007; Judd et al. 2005a,b; Guo et al. 2007), but differ from a recent study that concluded that the VirB proteins localize to the cell periphery (Aguilar et al. 2010). However, in the later study bacteria with VirB4-GFP or VirB11-GFP, partially functional in DNA transfer, did exhibit strong foci of fluorescence, and several of the foci mapped to a cell pole (see Fig. S2, lanes I and K in Aguilar et al. 2010). The authors questioned these observations because of a dominant negative phenotype of the two fusions (Aguilar et al. 2010, 2011). In the present study, we demonstrated that a GFP-VirB11 fusion that localized to a cell pole is fully functional in DNA transfer and had no negative effect on the DNA transfer property of wild-type bacteria (Fig.2 and data not shown). GFP-VirB4 also did not have a dominant negative phenotype.

Both VirB4 and VirB11 are hexameric proteins. A dominant negative phenotype could result from the assembly of a hetero-oligomer of VirB4/B11 and VirB4-/B11-GFP. In the other study, the addition of GFP at the C-terminus of both VirB4 and VirB11 affected an essential DNA transfer function and rendered the hetero-oligomer nonfunctional in DNA transfer. Our results show that GFP tag at the VirB11 N-terminus has no negative effect on either polar localization or DNA transfer. A similar addition was detrimental to the DNA transfer function of VirB4.

Fusions of GFP and membrane proteins are known to mislocalize to cell poles. Three VirB4 fusions analyzed in this study (encoded in pADI15ΔR, pADI19, and pADI28) contain one or more MSD. None of these fusions, however, localized to a cell pole indicating that the mere presence of a MSD is not sufficient for polar targeting of VirB4 (Fig.4). Also, the smallest VirB4 polar targeting region, residues 643–789, is hydrophilic and contains no strong hydrophobic segment (Dang and Christie 1997). At least one VirB11 fusion that failed to localize to a cell pole, GFP-VirB11_2-150_, is very likely to be associated with the cell membranes because a previous study found that VirB11_1-157_ associates with the bacterial membranes (Rashkova et al. 1997). In other studies fusions of GFP and several integral membrane proteins, viz., *Caulobacter* McpA, *Pseudomonas aeruginosa* PilS, *Agrobacterium* VirB6, *Caulobacter* DivJ, and *Agrobacterium* VirB10 did not randomly localize to the cell poles (Boyd 2000; Alley 2001; Sciochetti et al. 2002; Judd et al. 2005b; P. Judd, unpubl. results).

GFP fusions when overexpressed in *E. coli* are sometime targeted inappropriately to the cell poles (Winkler et al. 2010; Landgraf et al. 2012). In the present analysis of 26 fusions, no correlation was found between the cellular level of a protein and polar localization (Figs.3 and 5). In addition, we did not find polar targeting of GFP-VirB4 and GFP-VIrB11 to be concentration dependent. Both fusion proteins were found at a cell pole within 6–10 h of induction, a time at which the fusion proteins could barely be detected by western blot assays (A. Das, unpubl. results).

The PLDs of several proteins including *Shigella* IscA, *Caulobacter* McpA, DivJ and PopZ, *Pseudomonas* PilS, *B. subtilis* ClpC, and *Eubacterium rectale* TadZ have been mapped (Boyd 2000; Alley 2001; Charles et al. 2001; Sciochetti et al. 2002; Kain et al. 2008; Xu et al. 2012; Bowman et al., 2013). *Agrobacterium* VirB4 and *Shigella* IscA are the only two proteins in which two discontinuous regions function in polar targeting. The two IscA regions, however, share considerable sequence similarities (Charles et al. 2001). No sequence similarity within the three VirB4 and VirB11 PLDs could be identified.

Several mechanisms for the targeting of a protein to a cell pole have been identified (Dworkin 2009; Laloux and Jacobs-Wagner 2014). The *E. coli* proline transporter/osmosensor ProP localizes to the cell poles through its interaction with cardiolipin found at the rounded cell pole surface (Romantsov et al. 2007). The deletion of an *Agrobacterium* cardiolipin biosynthetic gene *cls* (Atu2486), however, had no effect on polar localization of GFP-VirD4 (A. Das, unpubl. results). The *C. crescentus* TipN protein marks the new pole and is required for proper localization of proteins and organelles (Huitema et al. 2006; Lam et al. 2006). *Bacillus subtilis* SpoVM directly recognizes the curvature of a membrane to localize to the forespore membrane, and not to the cytoplasmic membrane (Ramamurthi et al. 2009). *Bacillus subtilis* DivIVA protein is believed to contain a polar targeting sequence that recognizes some physical aspect of the pole, for example, curvature, to take it to the cell pole (Lenarcic et al. 2009; Ramamurthi et al. 2009). Protein–protein interactions are postulated to be required for polar targeting of proteins, for example, the social mobility protein FrsZ and the pilus assembly protein TadZ (Xu et al. 2012). Future studies will determine whether interactions of the VirB4/VirB11 PLDs are required for their polar targeting.

## Supporting Information

Table S1 Primers used for the amplification of *virB4* and *virB11* gene segments by PCR.

## References

[b1] Aguilar J, Zupan J, Cameron TA, Zambryski PC (2010). *Agrobacterium* type IV secretion system and its substrates form helical arrays around the circumference of virulence-induced cells. Proc. Natl. Acad. Sci. USA.

[b2] Aguilar J, Cameron TA, Zupan J, Zambryski P (2011). Membrane and core periplasmic *Agrobacterium tumefaciens* virulence type IV secretion system components localize to multiple sites around the bacterial perimeter during lateral attachment to plant cells. mBio.

[b3] Alley MRK (2001). The highly conserved domain of the *Caulobacter* McpA chemoreceptor is required for its polar localization. Mol. Microbiol.

[b4] Atmakuri K, Ding Z, Christie PJ (2003). VirE2, a type IV secretion substrate, interacts with the VirD4 transfer protein at cell poles of *Agrobacterium tumefaciens*. Mol. Microbiol.

[b5] Atmakuri K, Cascales E, Christie PJ (2004). Energetic components VirD4, VirB11 and VirB4 mediate early DNA transfer reactions required for bacterial type IV secretion. Mol. Microbiol.

[b6] Atmakuri K, Cascales E, Burton OT, Banta LM, Christie PJ (2007). *Agrobacterium* ParA/MinD-like VirC1 spatially coordinates early conjugative DNA transfer reactions. EMBO J.

[b7] Berger B, Christie P (1994). Genetic complementation analysis of the *Agrobacterium tumefaciens virB* operon: *virB2* through *virB11* are essential virulence genes. J. Bacteriol.

[b8] Berkman M, Lee C, Loveday E-K, Grossman A (2010). Polar positioning of a conjugation protein from the integrative and conjugative element ICEBs1 of *Bacillus subtilis*. J. Bacteriol.

[b9] Bowman G, Lyuksyutova A, Shapiro L (2011). Bacterial polarity. Curr. Opin. Cell Biol.

[b100] Bowman G, Perez A, Ptacin J, Ighodaro E, Folta-Stogniew E, Comolli L, Shapiro L (2013). Oligomerization and higher-order assembly contribute to sub-cellular localization of a bacterial scaffold. Mol. Microbiol.

[b10] Boyd JM (2000). Localization of the histidine kinase PilS to the poles of *Pseudomonas aeruginosa* and identification of a localization domain. Mol. Microbiol.

[b11] Brown PJB, de Pedro MA, Kysela DT, Van der Henst C, Kim J, De Bolle X (2012). Polar growth in the alphaproteobacterial order Rhizobiales. Proc. Natl. Acad. Sci. USA.

[b12] Charles M, Perez M, Kobil J, Goldberg M (2001). Polar targeting of *Shigella* virulence factor IcsA in *Enterobacteriacae* and *Vibrio*. Proc. Natl. Acad. Sci. USA.

[b13] Christie PJ, Atmakuri K, Krishnamoorthy V, Jakubowski S, Cascales E (2005). Biogenesis, architecture, and function of bacterial type IV secretion systems. Annu. Rev. Microbiol.

[b14] Dang T, Christie PJ (1997). The VirB4 ATPase of *Agrobacterium tumefaciens* is a cytoplasmic membrane protein exposed at the periplasmic surface. J. Bacteriol.

[b15] Dworkin J (2009). Cellular polarity in prokaryotic organisms. Cold Spring Harb. Prespect. Biol.

[b16] Fronzes R, Schäfer E, Wang L, Saibil HR, Orlova EV, Waksman G (2009). Structure of a type IV secretion system core complex. Science.

[b17] Guo M, Jin S, Sun D, Hew CL, Pan SQ (2007). Recruitment of conjugative DNA transfer substrate to *Agrobacterium* type IV secretion apparatus. Proc. Natl. Acad. Sci. USA.

[b18] Hinz A, Larson D, Smith C, Brun Y (2003). The *Caulobacter crescentus* polar organelle development protein PodJ is differentially localized and is required for polar targeting of the PleC development regulator. Mol. Microbiol.

[b19] Huitema E, Pritchard S, Matteson D, Radhakrishnan S, Viollier P (2006). Bacterial birth scar proteins mark future flagellum assembly site. Cell.

[b20] Judd PK, Kumar RB, Das A (2005a). Spatial location and requirements for the assembly of the *Agrobacterium tumefaciens* type IV secretion apparatus. Proc. Natl. Acad. Sci. USA.

[b21] Judd PK, Kumar RB, Das A (2005b). The type IV secretion apparatus protein VirB6 of *Agrobacterium tumefaciens* localizes to a cell pole. Mol. Microbiol.

[b22] Kain J, He GG, Losick R (2008). Polar localization and compartmentalization of ClpP proteases during growth and sporulation in *Bacillus subtilis*. J. Bacteriol.

[b23] Kumar RB, Das A (2002). Functional domains and polar location of the *Agrobacterium tumefaciens* DNA transfer protein VirD4. Mol. Microbiol.

[b24] Lai E-M, Chesnokova O, Banta L, Kado C (2000). Genetic and environmental factors affecting T-pilin export and T-pilus biogenesis in relation to flagellation of *Agrobacterium tumefaciens*. J. Bacteriol.

[b25] Laloux G, Jacobs-Wagner C (2014). How do bacteria localize proteins to the cell pole?. J. Cell Sci.

[b26] Lam H, Schofield W, Jacobs-Wagner C (2006). A landmark protein essential for establishing and perpetuating the polarity of a bacterial cell. Cell.

[b27] Landgraf D, Okumus B, Chien P, Baker TA, Paulsson J (2012). Segregation of molecules at cell division reveals native protein localization. Nat. Methods.

[b29] Lenarcic R, Halbedel S, Visser L, Shaw M, Wu LJ, Errington J (2009). Localisation of DivIVA by targeting to negatively curved membranes. EMBO J.

[b30] Llosa M, Roy C, Dehio C (2009). Bacterial type IV secretion systems in human disease. Mol. Microbiol.

[b31] Loh JT, Ho SC, de Feijter AW, Wang JL, Schindler M (1993). Carbohydrate binding activities of *Bradyrhizobium japonicum*: unipolar localization of the lectin BJ38 on the bacterial cell surface. Proc. Natl. Acad. Sci. USA.

[b32] Maddock JR, Shapiro L (1993). Polar location of the chemoreceptor complex in the *Escherichia coli* cell. Science.

[b33] Matthysse A (1987). Characterization of nonattaching mutants of *Agrobacterium tumefaciens*. J. Bacteriol.

[b34] Mossey P, Hudacek A, Das A (2010). *Agrobacterium tumefaciens* type IV secretion protein VirB3 is an inner membrane protein and requires VirB4, VirB7, and VirB8 for stabilization. J. Bacteriol.

[b35] Ramamurthi KS, Lecuyer S, Stone HA, Losick R (2009). Geometric cue for protein localization in a bacterium. Science.

[b36] Rashkova S, Spudich G, Christie PJ (1997). Characterization of membrane and protein interaction determinants of the *Agrobacterium tumefaciens* VirB11 ATPase. J. Bacteriol.

[b37] Romantsov T, Helbig S, Culham D, Gill C, Stalker L, Wood JM (2007). Cardiolipin promotes polar localization of osmosensory transporter ProP in *Escherichia coli*. Mol. Microbiol.

[b38] Schmidhauser T, Helinski D (1985). Regions of broad-host-range plasmid RK2 involved in replication and stable maintenance in nine species of gram-negative bacteria. J. Bacteriol.

[b39] Sciochetti SA, Lane T, Ohta N, Newton A (2002). Protein sequences and cellular factors required for polar localization of a histidine kinase in *Caulobacter crescentus*. J. Bacteriol.

[b40] Teng W, Bannam T, Parsons J, Rood J (2008). Functional characterization and localization of the TcpH conjugation protein from *Clostridium perfringens*. J. Bacteriol.

[b41] Vieira J, Messing J (1991). New pUC-derived cloning vectors with different selectable markers and DNA replication origins. Gene.

[b42] Vincent CD, Friedman JR, Jeong KC, Sutherland MC, Vogel JP (2012). Identification of the DotL coupling protein subcomplex of the *Legionella* Dot/Icm type IV secretion system. Mol. Microbiol.

[b43] Winkler J, Seybert A, Kig L, Pruggnaller S, Haselmann U, Sourjik V (2010). Quantitative and spatio-temporal features of protein aggregation in *Escherichia coli* and consequences on protein quality control and cellular ageing. EMBO J.

[b44] Xu Q, Christen B, Chiu H-J, Jaroszewski L, Klock HE, Knuth MW (2012). Structure of the pilus assembly protein TadZ from *Eubacterium rectale:* implications for polar localization. Mol. Microbiol.

[b45] Yeo HJ, Savvides SN, Herr AB, Lanka E, Waksman G (2000). Crystal structure of the hexameric traffic ATPase of the *Helicobacter pylori* type IV secretion system. Mol. Cell.

[b46] Zhang J, Campbell R, Ting A, Tsien R (2002). Creating new fluorescent probes for cell biology. Nat. Rev. Mol. Cell Biol.

[b47] Zupan J, Muth T, Draper O, Zambryski P (2000). The transfer of DNA from *Agrobacterium tumefaciens* into plants: a feast of fundamental insights. Plant J.

