# Supplementary material for: Delineation of polar localization domains of Agrobacterium tumefaciens type IV secretion apparatus proteins VirB4 and VirB11
Source: Microbiologyopen. 2014 Sep 13;3(5):793–802. doi: 10.1002/mbo3.208 (PMC4234268; doi:10.1002/mbo3.208)
Supplement: Table S1 — Primers used for the amplification of virB4 and virB11 gene segments by PCR. [file mbo30003-0793-sd1.docx]

**Supplementary Table**

Table 1. Primers used for the amplification of *virB4* and *virB11* gene segments by PCR.

| **Gene and Region**  **(codons) Amplified** | **Primers**  **FP, Forward Primer; RP, Reverse Primer** |
| --- | --- |
| **VirB4** |  |
| 2-236 | FP: AAAAGGATCCCTCGGCGCGAGTGGAAC  RP: AAAAGGTACCTCAAGGGCGTGTGCGCCACTT |
| 236-470 | FP: GACAGTTACGTTGGATCCATC  RP: AAAAGGTACCTCAGCCACCCCGGTCCTTGTC |
| 413-614 | FP: TTTTGGATCCGGCGGAACGCCTTTCGAC  RP: TTTTGGTACCTCATGCTGCAGCGGGAGCGCA |
| 562-789 | FP: TTTTGGATCCGAAGGCGCAGGAGCGC  RP: TTTTGGTACCGCGTCGTCTTCATCGTTTCC |
| 275-444 | FP: AAAAGGATCCCCTCAAGCGCACGCGAAA  RP: AAAAGGTACCTCACATCATGAGCGTTGTCTTACCCC |
| 275-376 | FP: AAAAGGATCCCCTCAAGCGCACGCGAAA  RP: AAAAGGTACCTCAAGGGCGTGTGCGCCACTT |
| 345-444 | FP: AAAAGGATCCGCGGATGCAGGTGCCGT  RP: AAAAGGTACCTCACATCATGAGCGTTGTCTTACCCC |
| 236-444 | FP: GACAGTTACGTTGGATCCATC  RP: AAAAGGTACCTCACATCATGAGCGTTGTCTTACCCC |
| 236-376 | FP: GACAGTTACGTTGGATCCATC  RP: AAAAGGTACCTCAAGGGCGTGTGCGCCACTT |
| 236-561 | FP: GACAGTTACGTTGGATCCATC  RP: AAAAGGTACCTCAGGCAGGGCCATGCAAC |
| 275-470 | FP: AAAAGGATCCCCTCAAGCGCACGCGAAA  RP: AAAAGGTACCTCAGCCACCCCGGTCCTTGTC |
| 345-470 | FP: AAAAGGATCCGCGGATGCAGGTGCCGT  RP: AAAAGGTACCTCAGCCACCCCGGTCCTTGTC |
| 592-789 | FP: TTTTGGATCCCCGTCGATTACCGGCTTC  RP: TTTTGGTACCGCGTCGTCTTCATCGTTTCC |
| 592-721 | FP: TTTTGGATCCCCGTCGATTACCGGCTTC  RP: TTTTGGTACCTCACGTCATGTCTTCACGGATCGC |
| 693-789 | FP: TTTTGGATCCCCATCACCAACCGCAGATCG  RP: TTTTGGTACCGCGTCGTCTTCATCGTTTCC |
| 592-755 | FP: TTTTGGATCCCCGTCGATTACCGGCTTC  RP: TTTTGGTACCTCAACGCCCCGAAAGTACGGCGAC |
| 643-755 | FP: TTTTGGATCCCCTAAATTTTCGGCCGTCGTCGAC  RP: TTTTGGTACCTCAACGCCCCGAAAGTACGGCGAC |
| 643-789 | FP: TTTTGGATCCCCTAAATTTTCGGCCGTCGTCGAC  RP: TTTTGGTACCTCAACGCCCCGAAAGTACGGCGAC |
| **VirB11** |  |
| 2-217 | FP: TTTTGGATCCGAAGTCGATCCGCAACTACGCTTT  RP: TTTTGGTACCTCAACCGTTCTTTGAGTAGAGTAGTC |
| 149-343 | FP: TTTTGGATCCGGGGATTTGGAAGCGTTTC  RP: GGGGTCTAGAGCTCGAGTTGATTTAGGAGATCGCC |
| 149-217 | FP: TTTTGGATCCGGGGATTTGGAAGCGTTTC  RP: TTTTGGTACCACCGTTCTTTGAGTAGAGTAGTC |
| 2-150 | FP: TTTTGGATCCGAAGTCGATCCGCAACTACGCTTT  RP: CCCGGTACCTCAATCCCCGTTGTCAAAATGCTG |
| 221-343 | FP: TTTTGGATCCGGTGCCGTGAGCGCC  RP: GGGGTCTAGAGCTCGAGTTGATTTAGGAGATCGCC |
| 149-248 | FP: TTTTGGATCCGGGGATTTGGAAGCGTTTC  RP: TTTTGGTACCTCAATCGTCGCGCATCTCGCCCAG |
| 123-248 | FP: TTTTGGATCCTCGAGGTGGAACCAGTGG  RP: TTTTGGTACCTCAATCGTCGCGCATCTCGCCCAG |
